# Temporal Characteristics of Stress Signals Using GRU Algorithm for Heavy Metal Detection in Rice Based on Sentinel-2 Images

**DOI:** 10.3390/ijerph19052567

**Published:** 2022-02-23

**Authors:** Yu Zhang, Meiling Liu, Li Kong, Tao Peng, Dong Xie, Li Zhang, Lingwen Tian, Xinyu Zou

**Affiliations:** 1 School of Information Engineering, China University of Geosciences, Beijing 100083, China; 2004190017@cugb.edu.cn (Y.Z.); 2004190016@cugb.edu.cn (T.P.); 2004190008@cugb.edu.cn (D.X.); 2004190014@cugb.edu.cn (L.Z.); tlw@cugb.edu.cn (L.T.); 3004190001@cugb.edu.cn (X.Z.); 2 Urban and Rural Planning and Design Institute Co., Ltd., Anhui Jianzhu University, Hefei 230022, China; 18895311631@163.com

**Keywords:** remote sensing, heavy metal stress, GRU model, red edge, time series

## Abstract

Heavy metal stress, which is a serious environmental problem, affects both animal and human health through the food chain. However, such subtle stress information is difficult to detect in remote sensing images. Therefore, enhancing the stress signal is key to accurately identifying heavy metal contamination in crops. The aim of this study was to identify heavy metal stress in rice at a regional scale by mining the time-series characteristics of rice growth under heavy metal stress using the gated recurrent unit (GRU) algorithm. The experimental area was located in Zhuzhou City, Hunan Province, China. We collected situ-measured data and Sentinel-2A images corresponding to the 2019–2021 period. First, the spatial distribution of the rice in the study area was extracted using the random forest algorithm based on the Sentinel 2 images. Second, the time-series characteristics were analyzed, sensitive parameters were selected, and a GRU classification model was constructed. Third, the model was used to identify the heavy metals in rice and then assess the accuracy of the classification results using performance metrics such as the accuracy rate, precision, recall rate (recall), and F1-score (F1-score). The results showed that the GRU model based on the time series of the red-edge location feature index has a good classification performance with an overall accuracy of 93.5% and a Kappa coefficient of 85.6%. This study shows that regional heavy metal stress in crops can be accurately detected using the GRU algorithm. A combination of spectrum and temporal information appears to be a promising method for monitoring crops under various types of stress.

## 1. Introduction

Heavy metal stress (e.g., Cuprum, Cadmium, and Zinc) in rice is one of the most serious types of farmland pollution. Accurate identification and monitoring of such stress can not only provide reasonable suggestions for the safe production of rice, but also protect human health to some extent. Remote sensing technology represents an efficient and economical means for monitoring and assessing heavy metal pollution in rice on a large scale [1]. However, in natural farmland ecosystems, changes in the physiological indicators of crop growth appear similar under different types of stresses (heavy metal stress, pest, and disease stress), making it difficult to accurately identify heavy metal stress [2,3]. Therefore, an accurate extraction of the heavy metal stress in rice remains challenging.

The spatiotemporal characteristics of crops under different stress conditions differ [4]. Generally, crops are mainly affected by stable and abrupt stresses during their growth [2,5]. Among them, abrupt stresses (e.g., drought, pests, and diseases) only occur in one or several growth cycles of the crop [2] and have different characteristics in different growth stages and across time [6]. In contrast, stable stressors (e.g., heavy metal stress) typically exist throughout the entire growth cycle of the crop and have a continuous long-term effect on crop growth. Heavy metal stress, which is a stable stress, has even more characteristics of low spatial mobility and stable presence over time [4,7]. Therefore, it is feasible to monitor the spatial distribution of heavy metals in farmlands based on multitemporal remote sensing images.

In the application of remote sensing time-series data, Tian et al. (2017) in [2] used an integrated empirical modal decomposition (EEMD) approach based on leaf area index (LAI) assimilation data to obtain the signal characteristics of heavy metal stress on long time scales, further demonstrating the potential of time-series data in improving the accuracy of heavy metal stress identification in crops. However, the method relies excessively on regionalized parameters, which must be reacquired for different regions, and the robustness of the model is insufficient. Tang et al. (2020) in [8] introduced the dynamic time warping (DTW) algorithm to analyze the year-to-year dissimilarity and spatial patterns of heavy metal stress in rice. However, the approach proposed by Tang et al. (2020) in [8] assumes that a pixel is affected by only a single stress and does not consider its exposure to the compound stress. To overcome these limitations, a deep learning method based on the temporal characteristics of the stress signals is introduced in this study.

Deep learning algorithms have the advantages of wide coverage, high learning ability, and portability, and are useful for mining the rich intrinsic information contained in images [9]. In terms of heavy metal stress identification, the deep learning algorithm does not require too many parameters and is not limited by whether the crop is subject to complex stresses; therefore, it can be used to mine the subtle information of the complex stress due to the heavy metal. To extract the time-stable features of heavy metal stress, the gated recurrent unit (GRU) algorithm based on the temporal characteristics of the stress signal was used in this study. As a type of deep learning algorithm, the GRU was first proposed in 2014 by Cho et al. [10]; in this method, the flow of information is controlled through gates that can be learned, which not only better capture the dependencies with large time steps in a time series, but also effectively solve the gradient explosion or gradient disappearance problem when capturing long-term dependencies using conventional recurrent neural networks (RNNs). Therefore, the advantages of the GRU can be fully exploited to identify heavy metal stress that is subtle, insidious, and temporally stable. In recent years, the GRU has been successfully applied in several fields, for example, the prediction of the time-series data of air pollutants [11,12], classification of spectral images [13,14,15,16], and the identification of vegetation health status [17]. However, the capability of the GRU method for classifying heavy metal stress in rice remains unexplored.

Therefore, the main contribution of this study was to combine deep learning algorithms with time series from the perspective of farmland health and to explore the application of GRU for heavy metal identification in rice based on the time-series characteristics of sensitive parameters at the red edge location.

## 2. Study Area and Data Processing

### 2.1. Study Area

**Figure 1:**
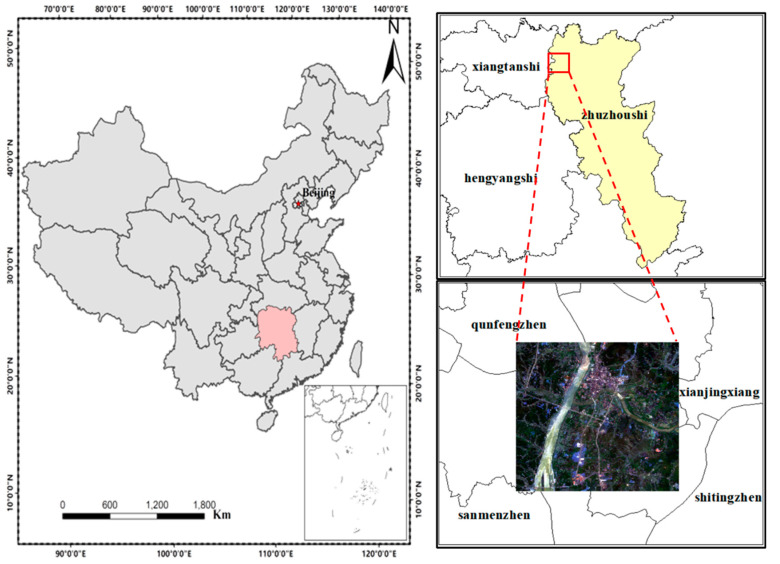
Location map for study areas in Zhuzhou, China.

The study area was located in Zhuzhou, Hunan Province, China (Figure 1), with latitude 24°3902″–29°4828″ N and longitude 110°1440″–110°4417″ E. The area has a subtropical monsoonal humid climate with four distinct seasons, abundant light, heat, and water resources, an annual average temperature range of 16–18 °C, and an annual average precipitation range of 1200–1700 mm [18]. The soil type is mainly red soil with a high organic matter content [3]. The superior climate and soil conditions have laid the foundation for high rice production; therefore, rice is an important cereal commodity in the region. The Xiangjiang River is the source of water for domestic, industrial, and agricultural use in Hunan. Considering that the main source of heavy metals in the Xiangjiang River basin is the nonferrous metal mining in Chenzhou and Hengyang, the long-term discharge of large amounts of industrial wastewater, waste gas, and sludge has exposed the soil, water, and crops in the Xiangjiang River basin to varying degrees of heavy metal pollution. Because Zhuzhou is located in the lower reaches of the Xiangjiang River basin, and studies have shown that many rice fields near the Xiangjiang River basin in Zhuzhou are seriously polluted by heavy metals, we selected a typical image area covering 1000 × 1000 pixels in Zhuzhou City as the study area based on previous research results and combined with actual rice measurement data.

### 2.2. Data Collection

#### 2.2.1. Sentinel-2 Satellite Images

In this study, we used images captured by the Sentinel-2 satellite, which is a high-resolution multispectral imaging satellite carrying a multispectral imager (MSI), commonly used for land monitoring [19]. It can provide monitoring information about agriculture and forestry cultivation and is important for predicting food production and ensuring food security. Sentinel-2 covers 13 spectral bands with a width of 290 km, from visible and near-infrared to short-wave infrared, with spatial resolutions of 10 m (B2, B3, B4, and B8), 20 m (B5–B7, B8a, B11, B12) and 60 m (B1, B9, B10) [20], with a revisit cycle of 10 days for one satellite and two complementary ones, with a revisit cycle of five days [21]. The Sentinel-2 image data in this study were mainly downloaded from the Google Earth Engine (GEE) platform (https://earthengine.google.com/) (accessed on 10 January 2022), and the Sentinel-2 products provided by the platform were divided into L1C products without atmospheric correction and L2A products with atmospheric correction. The dataset required for the study was composed of a single-scene multi-temporal images and based on the premise that the cloudiness was less than 30%. The spatial resolution of B2 to B12 bands was resampled to 10 m and downloaded locally, where the L2A products can be directly called for the data in 2019 and 2020, while the data in 2017 and 2018 are the ortho-corrected and sub-column geometrically corrected atmospheric apparent reflectance products Sentinel-2 Level-1C, which should be processed by Sen2cor. Sen2cor processing software was used to perform atmospheric correction operations on the data. Here, we selected product data corresponding to the 2019–2021 area.

#### 2.2.2. DEM Data

The Shuttle Radar Topography Mission (SRTM) digital elevation data were sorted in GEE, which helped produce digital elevation models (DEMs) on a near-global scale at a resolution of 30 m. The data show that rice is suitable for growth only on slopes less than 9° [22]. In this study, we collected this product to reduce the effects of slope and elevation on rice growth and then masked the areas where rice is unlikely to be grown.

#### 2.2.3. Verification Data

We used actual soil heavy metal samples (mainly Cadmium) and abandoned mine data as validation samples. The metal content in the soil was analyzed by the Chinese Academy of Agricultural Sciences in Beijing, China. Diethylene-triamine-pentaacetic acid (DTPA) extractable heavy metals in soil samples were analyzed using the method proposed by Lindsay and Norvell (1978) [23], and the metal concentrations were determined using an atomic absorption spectrophotometer (AAS) (SpectrAA 110/220, Varian, Palo Alto, CA, USA). The spatial distribution information of the abandoned mines in the study area was obtained from mineral resource statistics, including the China Important Mineral Database, National Bureau of Statistics, China National Coal Association, Metallurgical Mine Enterprises Association of China, and other departments (associations).

## 3. Methods

**Figure 2:**
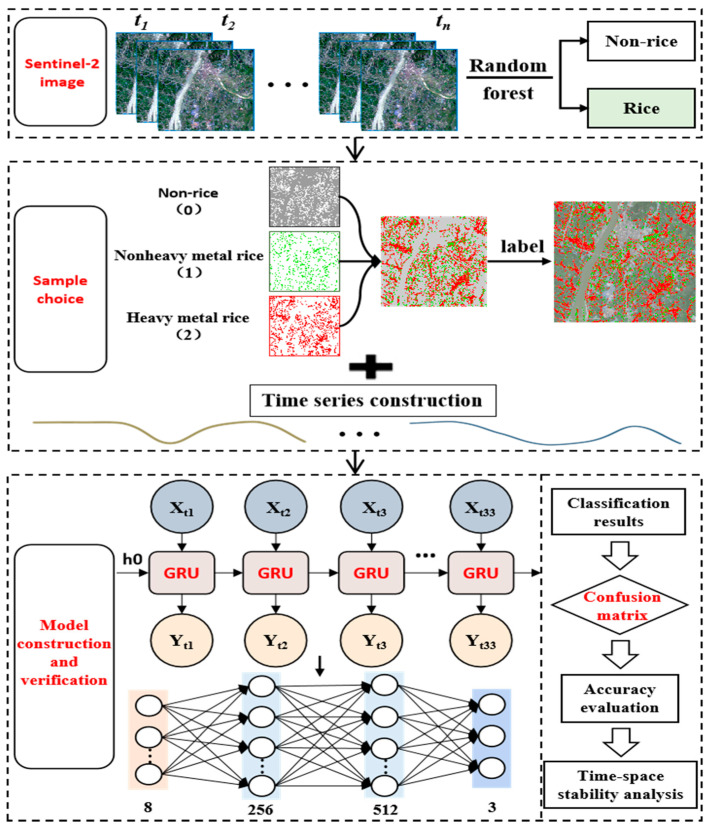
Flowchart of the GRU algorithm for the stress level monitoring of rice with heavy metal pollution.

This study focused on the identification and classification of heavy metal-stressed rice using a deep learning approach based on the GRU model. First, based on Sentinel-2 L2A images, the random forest [24] classification algorithm was used to obtain the extent of rice fields through the GEE platform. Second, a GRU-based heavy metal stress classification model for rice was constructed to extract the spatial distribution of image elements with heavy metal stress in the study area, and finally, the classification results were evaluated for accuracy. Figure 2 shows the workflow.

### 3.1. Extraction of Paddy Distribution

In this study, cloud-free Sentinel-2 images from April to mid-October, 2019–2021, were selected to map the spatial distribution of rice. We classified six land cover types (i.e., rice, forest, grass, water, urban, and bare land) using a random forest classifier. The training datasets were mainly obtained from field surveys and supplemented by visual interpretation using Google Earth imagery. The total training sample elements for the six land cover types were 1496 (1347 points, 149 surfaces), which were divided into two datasets: 70% training sample set and 30% test sample set. Finally, we used DEM data to generate slope information and removed image elements with slopes greater than 8° where rice would evidently not be grown in the range classification results, and further obtained a more accurate range for rice growth.

### 3.2. Detection of Heavy Metal Stress in Rice

#### 3.2.1. Selection of Parameters Sensitive to Heavy Metal Stress

Among optical data, the Sentinel-2 satellite image is the only one with three bands in the red-edge range, which is effective for monitoring vegetation health information [25]. Numerous studies have shown that stressed rice shows increased reflectance in the visible band, decreased reflectance in the near-infrared band, and shallowing of the “red valley” and blue shift of the “red edge” [26,27,28]. In recent years, spectral indices, such as the red edge position, red edge chlorophyll index, and normalized difference red-edge index, have been widely used for heavy metal stress monitoring in crops [29,30,31,32,33]. To improve the accuracy of identifying rice subjected to heavy metal stress, based on previous research results, a total of eight spectral parameters sensitive to heavy metal stress in rice were selected as classification indices in this study: Red-edge position (REP), red-edge chlorophyll index (${\mathrm{CI}}_{\mathrm{red}-\mathrm{edge}}$), modified simple ratio (MSR), modified chlorophyll absorption ratio index (MCARI), two normalized difference vegetation index (NDVI, RDVI), and two normalized difference red-edge indices (NDRE1, NDRE2), as shown in Table 1.

#### 3.2.2. Analysis of Temporal Characteristics of Stress Signal

**Figure 3:**
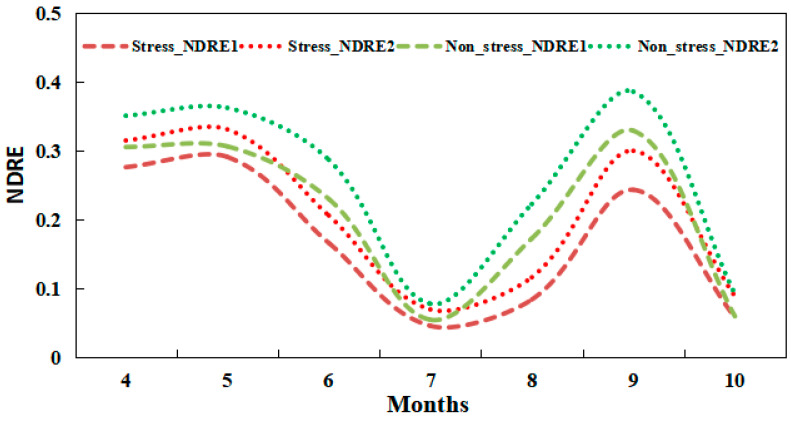
Temporal characteristics of stress signal from a single rice pixel under different stress types.

The temporal characteristics of the red-edge index series of rice under heavy metal stress were subtly different from those of unstressed rice. Taking the NDRE as an example, Figure 3 shows the time-series characteristics of the red-edge index of rice under the two stress conditions in our study area.

Throughout the growth period of rice, the trend in the NDRE sequences in heavy metal-stressed rice was similar to that in unstressed rice; however, their NDRE values were generally lower than those of the latter. In particular, the NDRE curves of rice subjected to heavy metal stress during June–August had troughs with greater spans. The deep learning approach can be used to mine these subtle differences in the temporal features and thus distinguish heavy metal stress.

#### 3.2.3. Establishment of Model for Detecting Heavy Metal Stress in Rice

**Figure 4:**
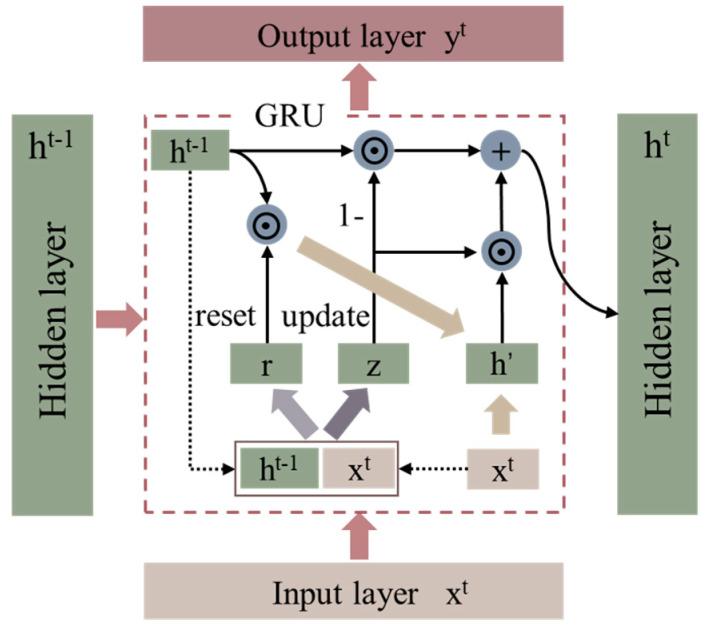
Schematic of GRU model.

As shown in Figure 4, the GRU comprises a reset gate and an update gate [10], which modifies the calculation of the hidden states in recurrent neural networks using a reset gate to discard historical information irrelevant to the prediction and help capture short-term dependencies in the time series. The update gate is used to capture the long-term dependencies in the time series. Here, the inputs of the gating are both $X_{t}$ of the current time step input and the hidden state $H_{t-1}$ of the previous time step, and the output is obtained by the fully connected layer of the activation function sigmoid function, calculated as:

(1) $$R_{t}=\sigma \left(X_{t}H_{\mathrm{xr}}+H_{t-1}W_{\mathrm{hr}}+b_{r}\right)$$

(2) $$Z_{t}=\sigma \left(X_{t}H_{\mathrm{xz}}+H_{t-1}W_{\mathrm{hz}}+b_{z}\right)$$

where $R_{t}$ and $Z_{t}$ are the computed results of the reset and update gates, respectively ($R_{t}\in R^{n\times h}$, $Z_{t}\in R^{n\times h}$), h is the number of hidden units, t is the given time step, $X_{t}\in R^{n\times d}$ (n is the number of samples, and d is the number of inputs) is the small batch input at time t; $H_{t-1}\in R^{n\times h}$ is the hidden state at the previous time step, and $W_{\mathrm{xr}},W_{\mathrm{xz}}\in R^{d\times h}$ and $W_{\mathrm{hr}},W_{\mathrm{hz}}\in R^{h\times h}$ are the weight parameters, and $b_{r},b_{z}\in R^{1\times h}$ are the deviation parameters.

The calculation of the hidden state requires the candidate hidden state, that is, the output of the reset gate of the current time step is multiplied by elements (⊙) with the hidden state of the previous step; and if the element in the reset gate is 0, the reset corresponding hidden state element is zero, which is considered as discarding the hidden state of the previous time step; in contrast, if the element in the reset gate is 1, the hidden state of the previous time step is retained [34]. In turn, the result of the element multiplication is concatenated with the input of the current time step, and the candidate hidden states are calculated by the fully concatenated layer of the activation function tanh.

(3) $${\tilde{H}}_{t}=\tanh(X_{t}W_{\mathrm{xh}}+\left(R_{t}⊙H_{t-1})W_{\mathrm{hh}}+b_{h}\right)$$

where ${\tilde{H}}_{t}\in R^{n\times h}$ is the candidate hidden state of time step t, $W_{\mathrm{xh}}\in R^{d\times h}$ and $W_{\mathrm{hh}}\in R^{h\times h}$ are the weight parameters, and $b_{h}\in R^{1\times h}$ is the deviation parameter.

The hidden state $H_{t}\in R^{n\times h}$ of time step t is finally calculated by combining the hidden state $H_{t-1}$ of the previous time step and the candidate hidden state ${\tilde{H}}_{t}$ of the current time step through the update gate $Z_{t}$ of the current time step.

When the number of time steps is large or the time series is small, the recurrent neural network is prone to gradient decay and gradient explosion. While the gradient explosion problem can be solved by cropping the gradient, the gradient decay problem cannot be solved. Therefore, this study uses the GRU to capture the dependencies with large time-step distances in the time series to further improve the training accuracy of the model. The model mainly comprises an input layer, a GRU layer, an output layer, two fully connected layers, and several hidden layers. Softmax regression is introduced to make the model to output more suitably for predicting and training the discrete values. Table 2 presents some of the parameters of the GRU model.

### 3.3. Detection of Heavy Metal Stress in Rice

We assessed the classification accuracy of paddy rice using both the confusion matrix and kappa coefficient. The confusion matrix is a common method used to evaluate the prediction results of the classification models in data analyses and machine learning. It has been used to compare the classification results with the actual measured values and record the correspondence between the true class of the samples and the predicted class of the model in the form of a matrix [35], where the accuracy rate, precision, recall rate (recall), and F1-score (F1-score) are chosen as evaluation metrics. Compared with the confusion matrix in which the objectivity of the overall accuracy, user accuracy, and producer accuracy is more dependent on the sample as well as the method, the Kappa coefficient is a more objective method to evaluate whether the classification is accurate or not; it uses a discrete multivariate method to determine to what degree the classification result is better than the result produced after random classification, thus avoiding the confusion matrix to be overly dependent on the sample and the process of collecting sample data [36,37].

(4) $${\mathrm{Accuracy}}_{i}=\frac{{\mathrm{TP}}_{i}+{\mathrm{TN}}_{i}}{{\mathrm{TP}}_{i}+{\mathrm{TN}}_{i}+{\mathrm{FP}}_{i}+{\mathrm{FN}}_{i}}$$

(5) $${\mathrm{Precision}}_{i}=\frac{{\mathrm{TP}}_{i}}{{\mathrm{TP}}_{i}+{\mathrm{FP}}_{i}}$$

(6) $${\mathrm{Recall}}_{i}=\frac{{\mathrm{TP}}_{i}}{{\mathrm{TP}}_{i}+{\mathrm{FN}}_{i}}$$

(7) $$F1\_{\mathrm{Score}}_{i}=\frac{{\mathrm{TP}}_{i}}{{\mathrm{TP}}_{i}+{\mathrm{FN}}_{i}}$$

The value range of i is 0–2, ${\mathrm{TP}}_{i}$ indicates the number of correct predictions of class i, ${\mathrm{TN}}_{i}$ indicates the number of correct predictions of the other classes, ${\mathrm{FP}}_{i}$ indicates the number of predictions of other classes as class i (wrong report); ${\mathrm{FN}}_{i}$ indicates the number of predictions of class i as other classes (missing report). The F1_Score metric combines the output results of Precision and Recall and takes a value in the range of 0–1, where 1 represents the best output result of the model, and 0 represents the worst output result.

## 4. Results

### 4.1. Spatial Distribution of Rice

**Figure 5:**
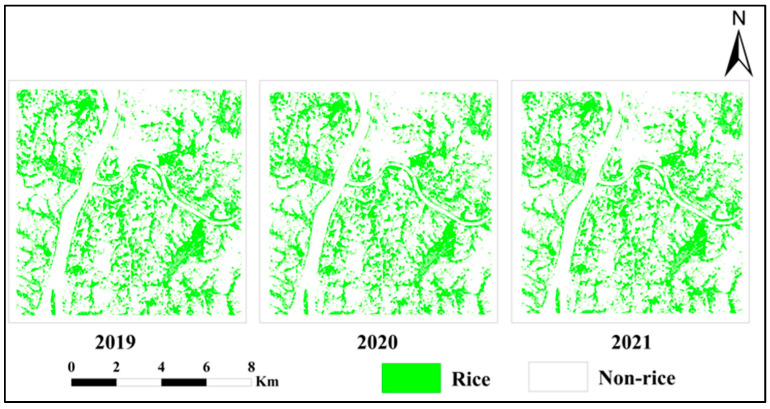
Spatial distribution of rice in the 2019–2021 period.

The spatial distribution of rice crops from 2019 to 2021 was obtained by applying the random forest classification method, and the overall accuracy of the land cover classification exceeded 92%, among which the user and producer accuracies exceeded 91%, and the Kappa coefficient exceeded 0.87. Figure 5 shows the rice planting range from 2019 to 2021, where no significant change can be seen in the spatial distribution of rice in the study area for three years.

### 4.2. Distribution of Heavy Metal Stress in Rice

**Figure 6:**
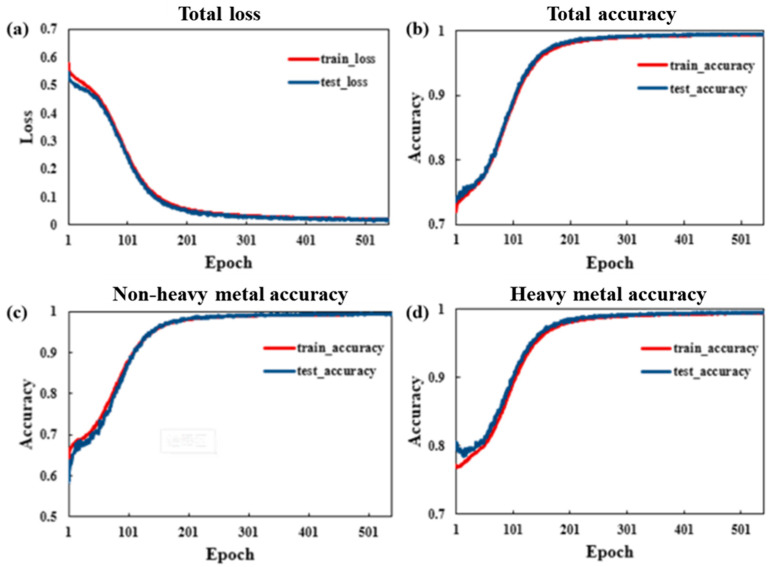
Accuracy and loss curves of the model (**a**): Overall Loss curve of the model. (**b**): Overall training and testing accuracy of the model. (**c**): Training and testing accuracy of non-heavy metal samples. (**d**): Training and testing accuracy of heavy metal samples.

In this study, a total of 33 cloud-free or cloud-less images covering the study area from April to October 2019 were selected, and the corresponding spectral indices were calculated, while the interference of cloud-contaminated pixels was removed using the QA60 band masking process. Based on the rice classification results, the rice samples under heavy metal stress, rice samples without heavy metal stress, and nonrice pixels were labeled separately. Only the rice image elements were classified by image element labeling, of which 153,300 pixels were classified as heavy metal-stressed rice samples and 79,918 pixels as nonheavy metal-stressed rice samples. The image data containing the feature sequences and the processed label data were inputted to the network model, and the sample data were divided into two groups: Training (80%) and testing (20%). Figure 6 shows the model training results.

As shown in Figure 6, the loss curve of the GRU model decreases smoothly and eventually stabilizes after several training sessions, and the trends in the train_loss and test_loss of the model are the same, indicating that the model has a better convergence effect; the overall accuracies of the model train_accuracy and test_accuracy are from the initial 50–60%, reaching 85–92% of the classification accuracy, respectively, from the initial interval of 50–60%, indicating that the model has a better overall classification effect. Figure 6c,d reflect the classification accuracies of rice without heavy metal stress and rice under heavy metal stress, respectively, and the accuracies on both the training and test sets of the model reached approximately 98%, demonstrating that the trained GRU model can distinguish each category well and meet the accuracy requirement of classification.

**Figure 7:**
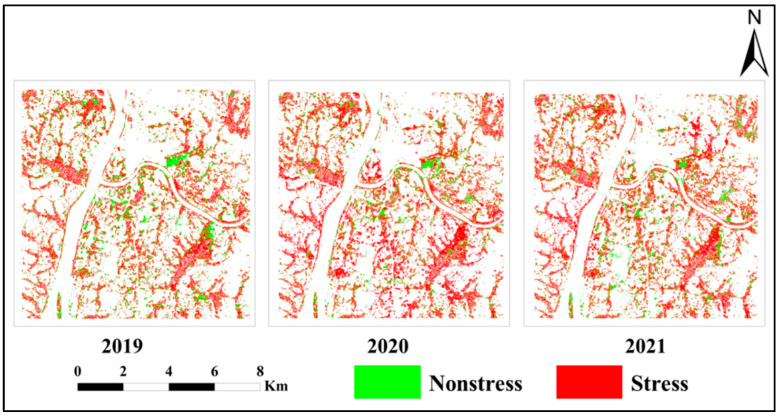
Spatial distribution of heavy metal-stressed rice from 2019 to 2021.

Based on the available Sentinel-2 images that were pre-processed during 2019–2021, eight characteristic index time series were generated and added to the GRU model for the classification of heavy metal-stressed rice in the study area. Figure 7 shows the spatial distribution of the heavy metal-stressed rice in each year, where white indicates nonrice areas, green indicates nonheavy metal-stressed rice, and red indicates heavily stressed rice.

The results showed that the spatial distributions of heavy metal stress in rice during 2019–2021 are similar and that they exhibit stable features in terms of the relative position in space. The areas that were not subjected to heavy metal stress were mostly located in the central part of the test area and the northeastern part, with a fragmented distribution, whereas the areas that were subjected to heavy metal stress were mainly concentrated in the northeastern, western, and southeastern parts of the study area, particularly in the form of a patchy distribution. Combined with the three-year classification results, this further confirmed that the heavy metal stress in rice is characterized by “spatial and temporal stability”.

### 4.3. Validation of Classification Results

#### 4.3.1. Accuracy Assessment of GRU Model

**Figure 8:**
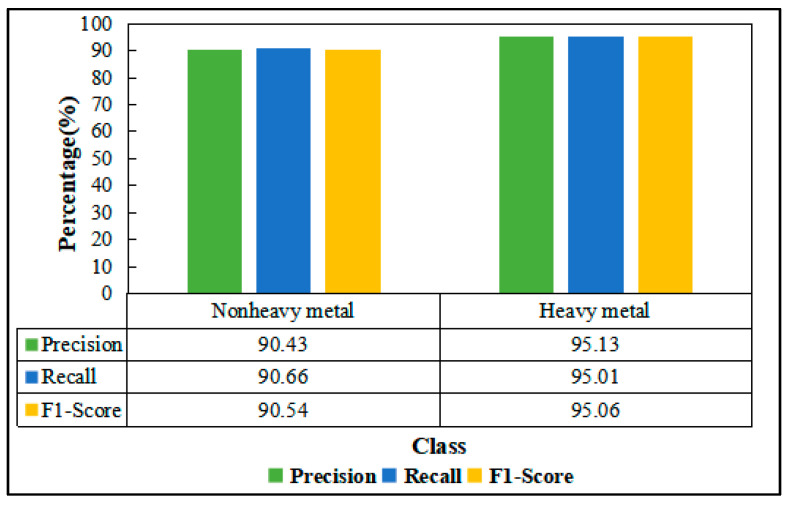
Classification accuracy in 2019.

The accuracy of the rice classification results was evaluated using the 2019 sample set in terms of the precision, recall, and F1-score, respectively. Figure 8 shows that the trained GRU model has a precision of 90.43%, recall of 90.66%, and F1-score of 90.54% for identifying nonheavy metal rice on the test set; the identification accuracy of rice under heavy metal stress was 95.12%, the recall rate was 95.01%, and the F1-score was 95.06%. This indicates that the model has a good classification recognition effect and that it meets the accuracy requirements for identifying rice under heavy metal stress in outdoor environments.

Because the spatial distribution of heavy metal stress in rice is “spatially and temporally stable”, the classification results for 2020 and 2021 were validated by randomly selecting the 2019 sample data based on the confusion matrix method; Table 3 and Table 4 show the validation results.

From Table 3 and Table 4, it can be seen that in 2020, the producer precision of the classification of rice without heavy metal stress is 86.87%, the user precision is 90.37%; the producer precision of the classification of rice with heavy metal stress is 95.30%, the user precision is 93.46%, the overall precision is 92.46%, and the Kappa coefficient is 82.96%. In 2021, for the classification of rice without heavy metal stress, the producer precision is 85.05%, user precision is 80.89%, producer precision is 93.28%, user precision is 94.91%, overall precision is 91.22%, and Kappa coefficient is 77.01%. This indicates that the GRU model based on the feature index time series can achieve good classification results.

#### 4.3.2. Relationship between Heavy Metal Pollution and Mining

**Figure 9:**
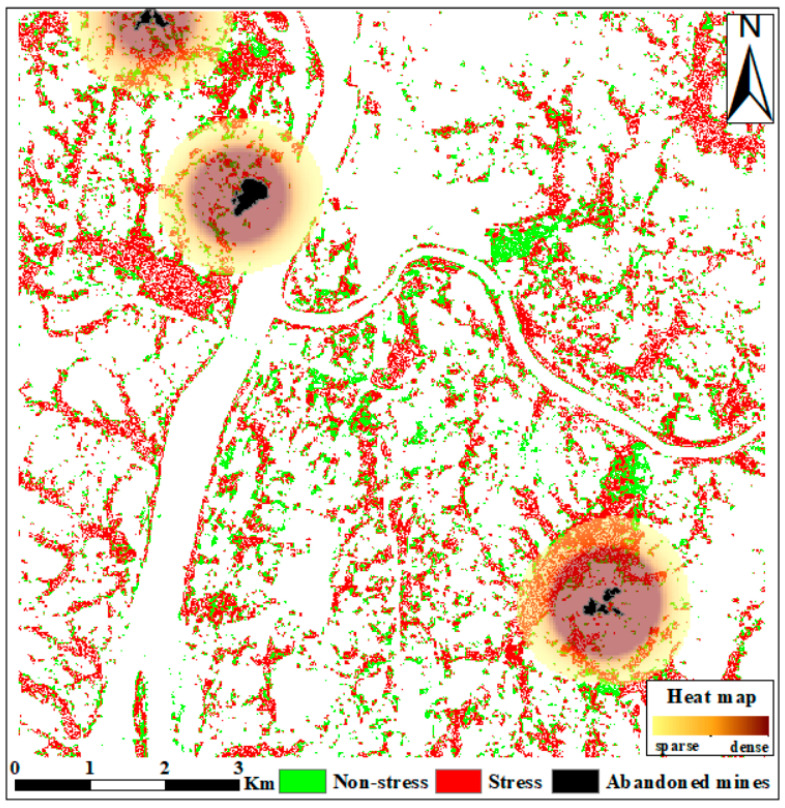
Distribution and heat map of abandoned mines in Zhuzhou.

To determine the relationship between heavy metal stress in rice and nonferrous metal mining, we obtained information on the spatial distribution of abandoned mines in the study area based on mineral resource statistics obtained from departments (associations) including the Chinese Important Mineral Mines Database, the National Bureau of Statistics, the Coal Industry Association, and the Metallurgical Mining Enterprises Association. The waste from mining ores contains toxic and harmful substances, which can cause organic toxic and heavy metal pollution to a large extent, which poses a threat to the surrounding soil and negatively affects the growth of rice. Figure 9 shows the distribution of abandoned mines in the study area.

The spatial distribution of the abandoned mines in Zhuzhou City shows the existence of three main mines in the northwestern and southeastern parts of the study area. Compared with the northwest area, the radiation contamination of the abandoned mines in the southeast is greater and is more likely to cause heavy metal contamination of rice. Moreover, there are patches of distributed rice fields near the mines, and the model classification results are consistent with the influence range of the mines.

## 5. Discussion

In this study, the time series of the red edge index was constructed, and the extraction of heavy metals from rice was realized based on the GRU algorithm. Good classification results were obtained. This is mainly because sensitive parameters were selected in the study [3], which can better reflect the stress information of heavy metal-contaminated rice. Second, compared to other stresses, heavy metal stress information was weaker. The GRU, which is a deep learning algorithm, could help mine the subtle differences in the feature sequences and extract this subtle stress information.

While the GRU algorithm can achieve better classification results, some limitations remain. First, we removed the influence of cloud noise based on the QA60 band, which may lead to a loss of some parts of the rice image elements covered by clouds and affect the classification accuracy. Second, the deep learning model relies on a large amount of sample data, and the selection of the model training samples is based on the measured data and previous research results for expansion, which may also bring some cumulative errors to the model. Furthermore, the current classification model is only applicable to a small area in the study area, and for applications at large spatial scales, the randomness of the cloud volume will largely affect the model classification results. Therefore, we will artificially add random noise in future studies to further improve the classification model and increase the robustness of the model and then classify and identify the heavy metals of rice in the entire Xiangjiang River basin.

## 6. Conclusions

This study explored the application of GRU-based deep learning algorithms for the classification of rice subjected to heavy metal stress in medium- and high-resolution images. First, cloud-free or cloud-less Sentinel-2 L2A images covering the entire rice growing period of 2019–2021 in the study area were screened to remove the effect of cloud noise. Second, sensitive parameters were selected, and a characteristic index time series was constructed. Finally, a GRU-based classification model was constructed, and the classification results were validated using a confusion matrix. The main findings and conclusions of this study are as follows:

(1) Heavy metal stress could be distinguished by extracting the time-series features of the red edge index of rice. This is because the red edge can reflect the health information of the crop, and the heavy metal stress signal is stable throughout the growth stage.
(2) The GRU model based on the characteristic index time series could achieve good classification results in identifying rice under heavy metal stress: Over 90% in 2019 and over 80% in 2020 and 2021.

In summary, the classification algorithm combined with deep learning can be used as a new means to detect heavy metal stress in rice and can provide a new idea for stress monitoring to prevent farmland pests and diseases.

## Figures and Tables

**Table 1:** Sensitive spectral parameters for monitoring heavy metal stress in rice.

Bands	Formula	Description
REP	700 + 40 * (((B7 − B4)/2 − B5)/(B6 − B5))	Red-edge position
${\mathrm{CI}}_{\mathrm{red}-\mathrm{edge}}$	(B7/B5) − 1	Red-edge chlorophyll index
MSR	(B6 − B1)/(B5 − B1)	Modified simple ratio
MCARI	((B5 − B4) − 0.2 * (B5 − B3)) * (B5/B4)	Modified chlorophyll Absorption ratio index
NDVI	(B8 − B4)/(B8 + B4)	Normalized difference Vegetation index
RDVI	$(B7-B4)/(\sqrt{B7+B4}$)	Renormalized difference Vegetation index
NDRE1	(B6 − B5)/(B6 + B5)	Normalized difference red-edge index 1
NDRE2	(B7 − B5)/(B7 + B5)	Normalized difference red-edge index 2

**Table 2:** Setting of GRU parameters.

Parameter	Value
Input_size	8
Hidden_size	256
Batch_size	8
Learning_rate	0.0001

where Input_size is the dimension of the input data, which represents the length of the feature vector; Hidden_size is the dimension of the hidden layer, which characterizes the number of nodes in each layer of the neural network, Batch_size is the number of sequence segment batches used for training, and Learning_rate is the hyperparameter that determines the training convergence speed.

**Table 3:** Classification accuracy evaluation in 2020.

Classified	Reference		Total	User’s Accuracy
	Nonheavy Metal	Heavy Metal		
Nonheavy metal	291	31	322	90.37%
Heavy metal	44	629	673	93.46%
Total	335	660	995	—
Producer’s Accuracy	86.87%	95.30%	—	—
Overall Accuracy 92.46%			Kappa 82.96%	

**Table 4:** Classification accuracy evaluation in 2021.

Classified	Reference		Total	User’s Accuracy
	Nonheavy Metal	Heavy Metal		
Nonheavy metal	182	43	225	80.89%
Heavy metal	32	597	629	94.91%
Total	214	640	854	—
Producer’s accuracy	85.05%	93.28%	—	—
Overall accuracy 91.22%			Kappa 77.01%	
